# Comprehensive Analysis of SLC17A9 and Its Prognostic Value in Hepatocellular Carcinoma

**DOI:** 10.3389/fonc.2022.809847

**Published:** 2022-07-25

**Authors:** Xue-Yan Kui, Yan Gao, Xu-Sheng Liu, Jing Zeng, Jian-Wei Yang, Lu-Meng Zhou, Xiao-Yu Liu, Yu Zhang, Yao-Hua Zhang, Zhi-Jun Pei

**Affiliations:** ^1^ Postgraduate Training Basement of Jinzhou Medical University, Taihe Hospital, Hubei University of Medicine, Shiyan, China; ^2^ Department of Nuclear Medicine and Institute of Anesthesiology and Pain, Taihe Hospital, Hubei University of Medicine, Shiyan, China; ^3^ Department of Infection Control, Taihe Hospital, Hubei University of Medicine, Shiyan, China; ^4^ Department of Nuclear Medicine, Xiangyang Cenral Hospital, Affiliated Hospital of Hubei University of Arts and Science, Xiangyang, China; ^5^ Department of Nuclear Medicine, Huanggang Central Hospital, Huanggang, China; ^6^ Hubei Key Laboratory of Embryonic Stem Cell Research, Shiyan, China

**Keywords:** SLC17A9, hepatocellular carcinoma, immune infiltration, m6A modification, TCGA, ferroptosis

## Abstract

**Background:**

Solute carrier family 17 member 9 (SLC17A9) encodes a member of a family of transmembrane proteins that are involved in the transport of small molecules. SLC17A9 is involved in the occurrence and development of various cancers, but its biological role in liver hepatocellular carcinoma (LIHC) is unclear.

**Methods:**

The expression level of SLC17A9 was assessed using The Cancer Genome Atlas (TCGA) database and immunohistochemistry of tumor tissues and adjacent normal liver tissues. The receiver operating characteristic (ROC) and R software package performed diagnosis and prognosis. Gene Ontology/Kyoto Encyclopedia of Genes and Genomes functional enrichment and co-expression of SLC17A9, gene–gene interaction (GGI), and protein–protein interaction (PPI) networks were performed using R, GeneMANIA, and STRING. Western blot, real-time quantitative PCR (RT-qPCR), immunofluorescence, colony formation, wound scratch assay, ATP production assays, and high connotation were applied to determine the effect of SLC17A9 knockdown on HEPG2 (hepatocellular liver carcinoma) cells. TIMER, GEPIA, and TCGA analyzed the relationship between SLC17A9 expression and immune cells, m6A modification, and ferroptosis.

**Results:**

SLC17A9 expression in LIHC tissues was higher than in normal liver tissues (p < 0.001), and SLC17A9 was related to sex, DSS (disease-specific survival), and PFI (progression-free interval) (p = 0.015, 0.006, and 0.023). SLC17A9 expression has diagnostic (AUC: 0.812; CI: 0.770–0.854) and prognostic potential (p = 0.015) in LIHC. Gene Ontology/Kyoto Encyclopedia of Genes and Genomes (GO/KEGG) functional enrichment analysis showed that SLC17A9 was closely related to neuronal cell body, presynapse, axonogenesis, PI3K/Akt signaling pathway. GGI showed that SLC17A9 was closely related to MYO5A. PPI showed that SLC17A9 was closely related to SLC18A3. SLC17A9 silencing inhibited HepG2 cells proliferation, migration, colony formation, and reduced their ATP level. SLC17A9 expression level was related to immune cells: B cells (r = 0.094, P = 8.06E-02), CD4^+^ T cells (r = 0.184, P = 5.95E-04), and macrophages (r = 0.137, P = 1.15E-02); m6A modification: HNRNPC (r = 0.220, p < 0.001), METTL3 (r = 0.180, p < 0.001), and WTAP (r = 0.130, p = 0.009); and ferroptosis: HSPA5 (r = 0.240, p < 0.001), SLC7A11 (r = 0.180, p < 0.001), and FANCD2 (r = 0.280, p < 0.001).

**Conclusion:**

Our data show that SLC17A9 may influence LIHC progression. SLC17A9 expression correlates with tumor immune infiltration, m6A modification, and ferroptosis in LIHC and may have diagnostic and prognostic value in LIHC.

## Introduction

Liver hepatocellular carcinoma (LIHC) is one of the most common malignant tumors in the world the most commonly diagnosed cancer in 13 countries and the leading cause of cancer death in 20 countries (1). Patients with advanced liver cancer often suffer liver failure. Liver cancer is mainly treated surgically, but it has high recurrence and metastasis. Its prognosis is very poor and its mortality rate is 8.2% (2). China has the heaviest burden of hepatitis and accounts for one in three global cases of chronic HBV infection and about 7% of HCV infections (3). Thus, effective liver cancer treatments are urgently needed.

SLC17A9 (solute carrier family 17 member 9), a vesicular nucleotide transporter (VUNT), is most abundantly expressed in the stomach, intestines, skeletal muscles, and liver (4). SLC17A9 is involved in ATP vesicular storage and exocytosis. SLC17A9 and its function were first described in bile duct cells (5). SLC17A9 is also highly enriched on lysosomes in various several cell types, including C2C12, COS-1, and HEK-293T cells, and actively transports ATP across lysosomal membranes (6). It is reported that ATP accumulates in lysosomes of astrocytes and microglia (7, 8). Once SLC17A9 is damaged, it will decrease in the accumulation of ATP in lysosomes, which results in cell death. SLC17A9 is expressed in biliary epithelial cells (9), osteoblasts (10), AR42J cells, and pancreatic acinar cells (11), but its specific role in LIHC has not been explored. Immunotherapy is a targeted method for the treatment of cancer. It is a common anti-tumor method to use the body’s immune system to fight tumor cells to promote the response to cancer cells (12, 13). Currently, m6A modification is widely used for the treatment of various cancers (14, 15). However, there are no studies on the role of SLC17A9 in LIHC, especially in the context of LIHC immunotherapy and m6A modification. Iron-dependent cell death has recently emerged as a novel form of cell death that occurs mainly due to an abnormal increase in intracellular iron-dependent lipid oxygen free radicals and an imbalance in redox homeostasis (16–18). However, little is known about the relationship between SLC17A9 and genes associated with iron-dependent cell death in LIHC. Here, we sought to characterize SLC17A9 expression in LIHC, its clinical significance, and its diagnostic and prognostic potential. We also examined the effect of SLC17A9 on the proliferation, migration, and colony formation capacity of LIHC cells and evaluated its biological function with the help of the database.

## Materials and Methods

### Database Analysis

Datasets on SLC17A9 expression in pan-cancer, LIHC, and normal liver tissues were downloaded from The Cancer Genome Atlas (TCGA) (https://portal.gdc.cancer.gov/) (19). Correlation between SLC17A9 expression in LIHC and clinicopathological features was analyzed using TCGA LIHC data. Receiver operating characteristic (ROC) curve analysis was done on R to detect the sensitivity and specificity of SLC17A9 expression in LIHC. Analysis of correlation between SLC17A9 expression and LIHC prognosis was done on R. LinkedOmics database (www.linkedomics.org/login.php) (20) was used to identify genes that are co-expressed with SLC17A9 in the TCGA LIHC dataset. Pearson’s correlation coefficient was used for statistical analysis. Volcano plots and heatmaps were used to visualize analysis results. An R package was used for GO and KEGG pathway enrichment analysis of SLC17A9 in LIHC. GeneMANIA (www.genemania.org) and STRING (www.string-db.org) were used to construct gene–gene interaction (GGI) and protein–protein interaction (PPI) networks (21, 22), respectively. TIMER (https://cistrome.shinyapps.io/timer) (23) was used to assess correlation between SLC17A9 gene expression and immune cell infiltration. TIMER’s SCNA module was used to associate SLC17A9’s genetic copy number variation (CNV) with the relative abundance of tumor-infiltrating cells. The proportion of immune cells in LIHC samples expressing high and low SLC17A9 levels was identified on R using the CiberSort plug-in. GEPIA (gepia.cancer-pku.cn) (24) was used to assess the relationship between SLC17A9 and immune cell markers in LIHC. Immune cell markers were obtained from the website of R&D Systems (www.rndsystems.com/cn/resources/cell-markers/immune-cells). Correlation between SLC17A9 and m6A and ferroptosis was analyzed using TCGA LIHC datasets.

### Liver Cancer Tissues Specimens

We recruited 28 patients with liver cancer who underwent surgery in Taihe Hospital, Shiyan City, Hubei province. Ethical approval for this study was granted by Taihe Hospital’s medical ethics committee. The study adhered to the Helsinki Declaration and its subsequent amendments.

### Cell Transfection

HepG2 cells were seeded on six-well plates at 6 × 10^5^ cells per well and cultured for 24 h. Transfection was done using Lipofectamine 8000 reagent (Beyotime) following the manufacturer’s guides. For each transfected well, 125 μl of serum-free OptiMEM, 2.5μg of plasmid DNA, and 4 μl of Lipo8000™ transfection reagent were mixed in a sterile centrifuge tube. The transfection mixture was applied to the cells. The oligonucleotide sequences of the small interfering RNA (siRNA) were displayed as follows: SLC17A9: (forward: 5′-CTTGCTCCAAGGGGTTTACTTC-3′, reverse: 5′-CCGGAGAAATAGAAGATGCTCT-3′); and GAPDH: (forward: 5′-CGCTGAGTACGTCGTGGAGTC-3′, reverse: 5 ′ GCTGATGATCTTGAGGCTGTTGTC-3′).

### Western Blot Analysis

At 48 h after transfection, cells were homogenized in RIPA lysis buffer (Promega, Madison, USA) on ice for 30 min, and then, protein was measured by the Bicinchoninic Acid (BCA) assay (Beyotime, Beijing, China). Protein was separated on 10% sodium dodecyl sulfate–polyacrylamide gels and transferred onto a polyvinylidene difluoride membrane using a semiwet method; next, we blocked the membrane with 5% dried skimmed milk for 1 h and incubated overnight with a SLC17A9 rabbit anti-human monoclonal antibody (1:200, Abcam, USA) in a 4°C refrigerator. Next day, horseradish peroxidase (HRP)–conjugated secondary antibodies (1:1,000; ProteinTech Group) for 1 h at room temperature. Images were obtained using a gel imaging system.

### RNA Isolation and Real-Time Quantitative PCR Analysis

Total RNA was extracted from cells 48 h after transfection using Trizol reagent (Invitrogen). Trizol Reagen (600 μl) was added, and the samples were placed on ice for 20 min to lyse the cells. Subsequently, the cell lysate in Tirol was phase separated by addition of 120 μl of chloroform, and the RNA was precipitated by addition of 300 μl of isopropanol, and then, cDNA was synthesized by utilizing the TaqMan Reverse Transcription Reagents kit (TaKaRa). Real-time quantitative PCR (RT-qPCR) analysis was performed with different primer sequences using the SYBR Green Master Mix (TaKaRa). The primers used for RT-PCR were in **Table 1**.

**Table 1:** Primer sequence (5′-3′).

Primer	Primer sequence (5′- 3′)
HNRNPC	Forward: CGTGTACCTCCTCCTCCTCCTATTG
	Reverse: CCCGCTGTCCACTCTTAGAATTGAAG
WTAP	Forward: CTGACAAACGGACCAAGTAATG
	Reverse: AAAGTCATCTTCGGTTGTGTTG
METTL3	Forward: CCAGCACAGCTTCAGCAGTTCC
	Reverse: GCGTGGAGATGGCAAGACAGATG
ACSL4	Forward: ACCAGGGAAATCCTAAGTGAAG
	Reverse: GGTGTTCTTTGGTTTTAGTCCC
CISD1	Forward: AACCTTCACATCCAGAAAGACAACCC
	Reverse: GACCTCCAACAACGGCAGTACAC
ATP5MC3	Forward: GGCTGGTTCTGGTGCTGGTATTG
	Reverse: AGCTTCAGACAAGGCAAATC

### Immunofluorescence Experiment

Cells were fixed with 4% formaldehyde for 1 h and permeabilized with 0.5% Triton X-100 at room temperature. They were then blocked with 1% Bovine Serum Albumin (BSA) for 2 h then incubated with primary antibody at 4°C, overnight. Next, they were incubated with secondary antibody 2 h and nuclei counterstained 2-(4-Amidinophenyl)-6-indolecarbamidine dihydrochloride (DAPI). They were then examined and imaged fluorescent confocal microscopy.

### Colony Formation Experiment

The cells were seeded onto 12-well plates at 100 cells per well and cultured for 2 weeks. They were then fixed with 4% Paraformaldehyde (PFA) for 10 min and rinsed with Phosphate Buffered Saline (PBS) before staining with crystal violet. They were then examined by microscopy and imaged.

### Wound Scratch Assay

HepG2 cells (1 × 10^6^ per well) were seeded onto 12-well plates and cultured until confluent. A white gun was used to make vertical scratch along the ruler to ensure uniform scratches in each well. The image was taken at 0, 12, 24, and 48 h.

### ATP Detection Assay

Control and siSLC17A9 groups cells were cultured for 72 h, and their culture media and cells were collected. Cellular ATP levels were then measured using an ATP bioluminescent assay kit (Beyotime, China).

### High Connotation Cell Imaging Analysis

Control and siSLC17A9 groups were suspended at 1 × 10^4^/ml and seeded onto 96-well plates in six replicate wells. After 24 h of culture, the 96-well plates were moved into a high-connotation instrument. High-connotation cell images were then taken using a 10× objective. Multiple visual fields were selected per well for monitoring and cell growth recording.

### Statistical Analysis

Gene expression analysis was done using Pearson correlation analysis. Other experimental results were analyzed using two-tailed t-test. Data were expressed as mean ± SD. P < 0.05 indicated statistically significant differences.

## Results

### SLC17A9 Is Highly Expressed in LIHC Tissues And Is Related to Gender, DSS, and PFI

**Figure 1:**
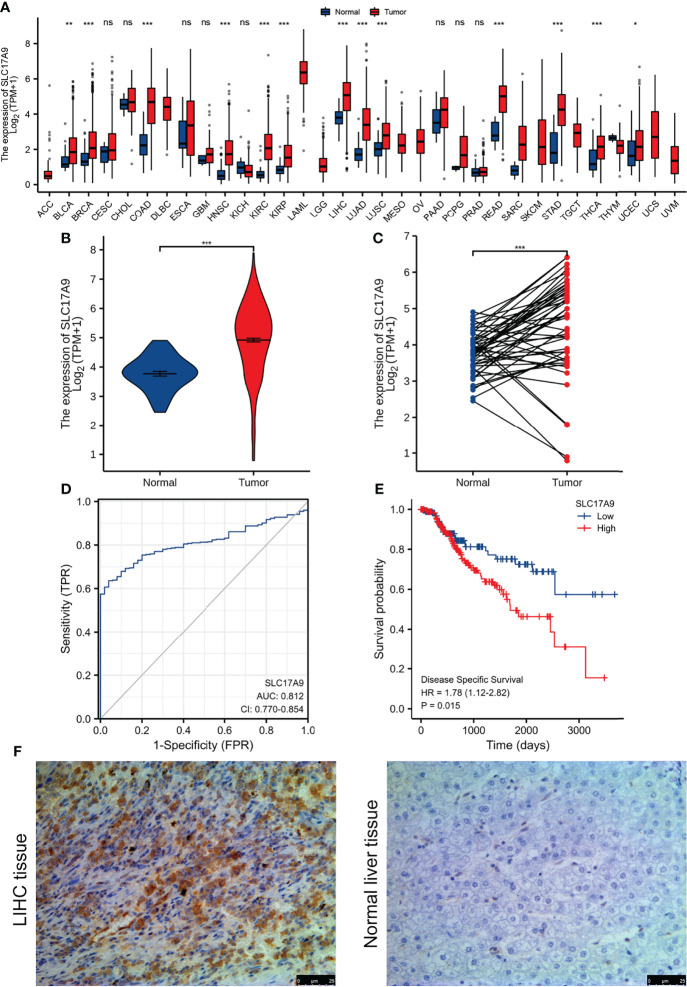
The expression of SLC17A9 in hepatocellular carcinoma (LIHC) and pan-carcinoma. **(A)** The TCGA database shows the SLC17A9 mRNA expression levels in different tumor types (Sample information: normal group, N = 730; and tumor group, N = 10,363). [The sample data of **(B–E)** are from TCGA-LIHC RNA-seq platform. Sample information: normal group, N = 50; and tumor group, N = 374]. **(B)** R software package was used to analyze SLC17A9 expression in non-paired samples between LIHC and normal tissues. **(C)** R software package was used to analyze the difference of SLC17A9 expression in paired samples between LIHC and normal tissues. **(D)** Receiver operating characteristic curve analysis showed that in predicting the outcome of normal and tumor groups, the predictive ability of variable SLC17A9 has a certain accuracy (AUC = 0.812; CI = 0.770–0.854). Sensitivity and specificity for the prediction of SLC17A9 expression were 63.6% and 96.0%, respectively. **(E)** R software package graph to show the survival analyses of patients with LIHC based on SLC17A9 expression. **(F)** Immunohistochemical staining for SLC17A9 in LIHC tissue and adjacent normal liver tissues. *p < 0.05; **p < 0.01; ***p < 0.001; ns, no significance.

We analyzed the expression level of SLC17A9 in TCGA pan-cancer dataset (**Figure 1A**). The results showed that SLC17A9 was highly expressed in 12 cancer types, including breast cancer, colonic adenocarcinoma, and LIHC. Low SLC17A9 expression was found in 21 cancers, including cervical cancer and cholangiocarcinoma. Analysis of SLC17A9 expression in LIHC vs. normal tissues in paired and unmatched samples revealed that SLC17A9 expression was significantly higher in LIHC tissues than in normal tissues (p < 0.001; **Figures 1B, C**). ROC analysis showed that the area under the ROC curve was 0.812. The sensitivity and specificity of predicting SLC17A9 expression in liver cancer were 63.6% and 96%, respectively (**Figure 1D**). Correlation analysis revealed that high SLC17A9 expression correlated with poor prognosis (**Figure 1E**). Relative to patients with low SLC17A9 expression, those with high SLC17A9 expression had significantly shorter survival (p = 0.015). Correlation analysis of TCGA data revealed correlation between SLC17A9 expression and some clinical features. High SLC17A9 expression correlated positively with gender, disease-specific survival (DSS), and progression-free interval (PFI) (p = 0.015, 0.006, and 0.023, respectively; **Table 2**). To confirm this finding, we carried out IHC analysis and found that SLC17A9 is mainly localized in the cytosol, with modest presence in the nucleus. IHC results showed that the protein level of SLC17A9 in tumor tissues was significantly higher than that in adjacent normal tissues (**Figure 1F**). These results indicate that SLC17A9 has a potential carcinogenic effect on the progression of LIHC.

**Table 2:** Correlation between SLC17A9 expressions and different clinicopathological characteristics in LIHC.

Characteristic	Levels	SLC17A9 Expression		P-Value
		Low (%)	High (%)	
Age	≤60	83 (22.3%)	94 (25.2%)	0.323
	>60	103 (27.6%)	93 (24.9%)	
Gender	Female	49 (13.1%)	72 (19.3%)	**0.015**
	Male	138 (36.9%)	115 (30.7%)	
T stage	T1	90 (24.3%)	93 (25.1%)	0.561
	T2	51 (13.7%)	44 (11.9%)	
	T3	36 (9.7%)	44 (11.9%)	
	T4	8 (2.2%)	5 (1.3%)	
N stage	N0	130 (50.4%)	124 (48.1%)	0.059
	N1	0 (0%)	4 (1.6%)	
M stage	M0	134 (49.3%)	134 (49.3%)	0.622
	M1	1 (0.4%)	3 (1.1%)	
Pathologic stage	Stage I	84 (24%)	89 (25.4%)	0.285
	Stage II	49 (14%)	38 (10.9%)	
	Stage III	39 (11.1%)	46 (13.1%)	
	Stage IV	1 (0.3%)	4 (1.1%)	
Histologic grade	G1	36 (9.8%)	19 (5.1%)	0.094
	G2	88 (23.8%)	90 (24.4%)	
	G3	57 (15.4%)	67 (18.2%)	
	G4	5 (1.4%)	7 (1.9%)	
OS event	Alive	125 (33.4%)	119 (31.8%)	0.587
	Dead	62 (16.6%)	68 (18.2%)	
DSS event	Alive	154 (42.1%)	133 (36.3%)	**0.006**
	Dead	28 (7.7%)	51 (13.9%)	
PFI event	Alive	107 (28.6%)	84 (22.5%)	**0.023**
	Dead	80 (21.4%)	103 (27.5%)	
Age, median (IQR)		62 (52, 69)	60 (51, 68)	0.286

Bold values indicate P < 0.05.

### Co-Expressed Genes of SLC17A9 in LIHC

**Figure 2:**
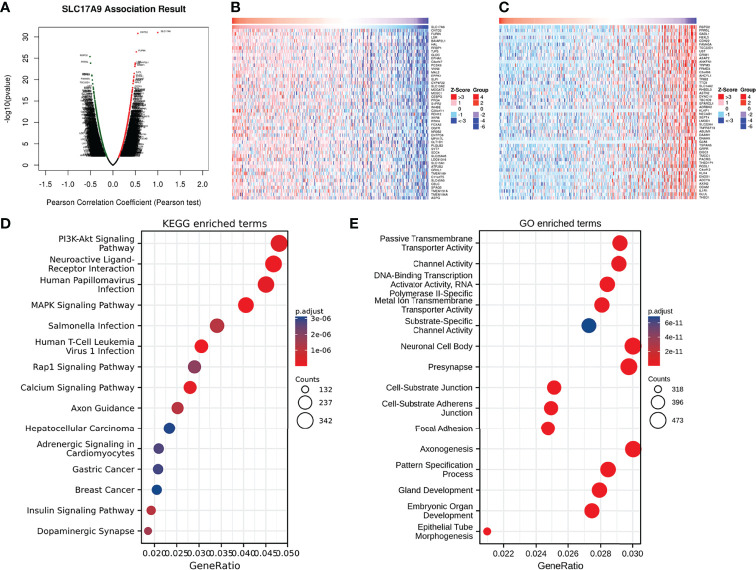
Enrichment analysis of SLC17A9 gene co-expression network in LIHC. **(A)** The volcano map showed co-expression genes associated with SLC17A9 expression in the TCGA LIHC data set. **(B)** Heatmaps showed the top 50 co-expression genes positively correlated with SLC17A9 expression in the LIHC. **(C)** Heatmaps showed the top 50 co-expression genes negatively correlated with SLC17A9 expression in the LIHC. **(D)** Enrichment of Gene Ontology (GO) SLC17A9 co-expression genes. **(E)** Enrichment of Kyoto Encyclopedia of Genes and Genomes (KEGG) terms for SLC17A9 co-expression genes.

Analysis of genes’ co-expression with SLC17A9 in LIHC on LinkedOmics found that SLC17A9 expression positively correlated with 4,084 genes and negatively correlated with 3,702 genes (**Figure 2A**). Heatmaps were used to visualize the top 50 genes that positively or negatively correlated with SLC17A9 expression, respectively (**Figures 2B, C**). GO/KEGG functional enrichment analysis (p < 0.05) of SLC17A9 identified 2,462 items related to biological process (GO-BP), 351 items related to cell component (GO-CC), 234 items related to molecular function (GO-MF), and 184 KEGG. KEGG pathway analysis showed that SLC17A9 co-expression was mainly associated with PI3K/Akt signaling pathway, neuroactive ligand-receptor interaction, Human Papillomavirus infection (HPV) infection, MAPK signaling, passive transmembrane transporter activity, and channel activity (**Figure 2D**). The bubble map GO function analysis showed that SLC17A9 co-expression was mainly associated with neuronal cell body, presynapse, and axonogenesis (**Figure 2E**).

### SLC17A9 -Related Hub Genes

**Figure 3:**
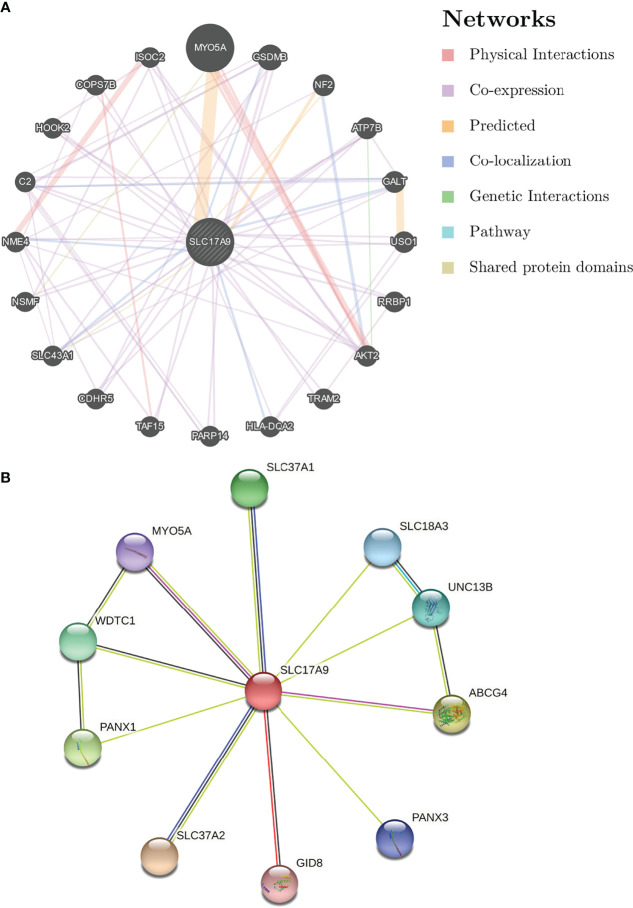
Analysis of gene–gene interaction (GGI) and protein–protein interaction (PPI) of SLC17A9. **(A)** GGI network of SLC17A9. **(B)** PPI interaction network of SLC17A9.

To understand the relationship between SLC17A9 expression in LIHC, a PPI network consisting of 20 nodes was constructed on the basis of the STRING database (**Figure 3A**). The nodes in the network show that genes were related to SLC17A9 in terms of physical interactions, co-expression, predicted, co-localization, genetic interactions, pathway, and shared protein domains. The gene with the most significant correlation with SLC17A9 was MYO5A, followed by GSDMB, NF2, ATP7B, and GALT. Prediction analysis showed that GSDMB and GALT genes were co-expressed and co-localized with SLC17A9. In addition, NF2 shared protein domains with SLC17A9, whereas ATP7B is co-expressed with SLC17A9.

PPI network analysis of SLC17A9 and related proteins was performed using the STRING webserver (**Figure 3B**). The results indicated that SLC17A9 was associated with SLC18A3 (vesicular acetylcholine transporter), PANX1 (pannexin-1), PANX3 (pannexin-3), MYO5A (unconventional myosin-Va), GID8 (glucose-induced degradation protein 8 homolog), ABCG4 (ATP-binding cassette sub-family G member 4), UNC13B (protein unc-13 homolog B), WDTC1 (WD and tetratricopeptide repeat protein 1), SLC37A1 (glucose-6-phosphate transporter member 1), and SLC37A2 (glucose-6-phosphate transporter member 2). The correlation coefficients were 0.947, 0.555, 0.518, 0.508, 0.506, 0.557, 0.551, 0542, 0.553, and 0.675, respectively.

### Effects of SLC17A9 Knockdown on the Function and ATP Levels of Hepatoma Cells

**Figure 4:**
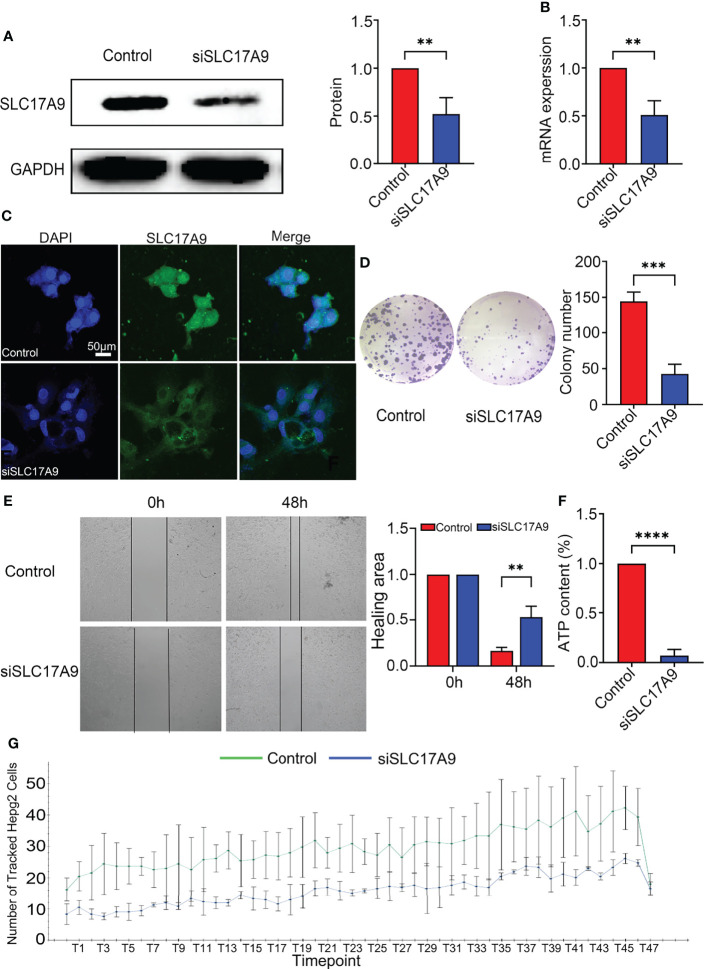
Silencing of SLC17A9 reduced malignant phenotypes of liver cancer. **(A)** Western blot showed the knockdown efficacy of SLC17A9 siRNA. **(B)** SLC17A9 mRNA expression level in control and siSLC17A9 groups. **(C)** Immunofluorescence detection of proliferation rate. **(D)** Colony formation assay results in control and siSLC17A9 groups. **(E)** Wound healing assay after transfection with SLC17A9 siRNA or control siRNA. **(F)** ATP levels were measured in the control and siSLC17A9 groups. **(G)** Using high-content imaging to detect the increment rate of cells in different periods. *p < 0.05; **p < 0.01; ***p < 0.001; ****p <0.0001.

Expression of SLC17A9 in HepG2 cells was down regulated by treatment of the cells with siRNA. Effectiveness of SLC17A9 knockdown in liver cancer cell line was determined by Western blot and RT-qPCR analysis that showed stable siSLC17A9 (**Figures 4A, B**). There was a significant difference in SLC17A9 expression level (P = 0.0083 and 0.002273) between siSLC17A9-related HepG2 cells and control HepG2 cells. Immunofluorescence assays showed that SLC17A9 was localized in the cell cytoplasm, and the expression level in the siSLC17A9 group was significantly lower relative to the expression level of SLC17A9 in the control group (**Figure 4C**). The colony-forming ability of cells after SLC17A9 knockdown showed a significant decrease relative the colony-forming ability of the control group (P = 0.0007; **Figure 4D**). Wound healing assay showed that the cell migration ability of the siSLC17A9 group was significantly reduced compared with the migration ability of the control group (P = 0.0069; **Figure 4E**). The results showed significantly lower HepG2 cell ATP content in the siSLC17A9 group relative to the ATP content in the control group (P < 0.0001; **Figure 4F**). Moreover, cells in the siSLC17A9 group showed a significant decrease in cell proliferation ability compared with the proliferate rate of cells in the control group (P < 0.05; **Figure 4G**).

### SLC17A9 Expression Level Is Correlated With Infiltration of Tumor Immune Cells

**Figure 5:**
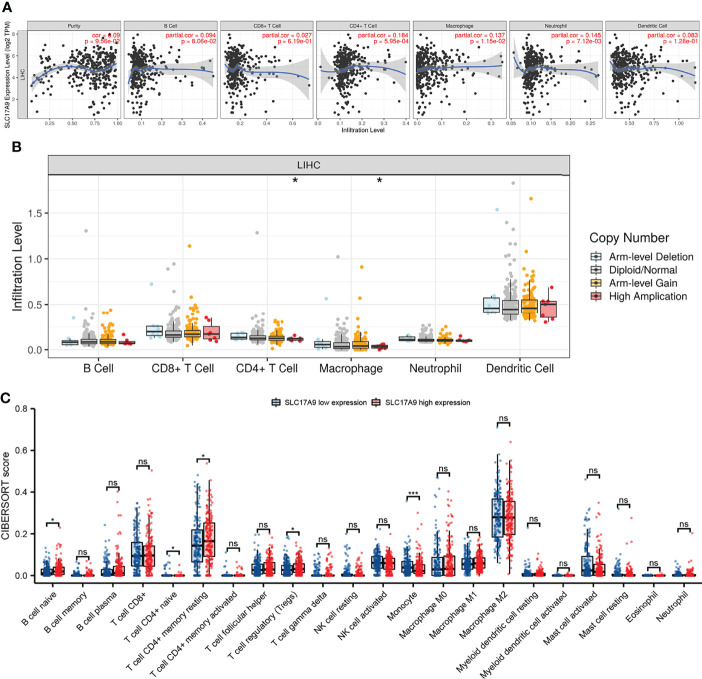
Analysis of the correlation between SLC17A9 and immune cells and genetic copy number variations (CNV) of SLC17A9 with the relative abundance of tumor-infiltrating cells and CIBERSORT analysis. **(A)** Correlation between SLC17A9 and tumor immune infiltrating cells **(B)** SLC17A9 CNV affects the infiltrating levels of CD4^+^ T cell and neutrophil cells in LIHC. **(C)** The change ratio of 22 immune cell subtypes in the high and low SLC17A9 expression groups in LIHC. *p < 0.05; **p < 0.01; ***p < 0.001; ****p < 0.0001.

The relationship between SLC17A9 and various immune cells in LIHC was explored using TIMER database. The findings showed that the expression level of SLC17A9 was correlated with infiltration of B cells (r = 0.094, P = 8.06E-02), CD4^+^ T cells (r = 0.184, P = 5.95E-04), macrophages (r = 0.137, P = 1.15E-02), neutrophils (r = 0.145, P = 7.12E-03), and CD8^+^ T cells (r = 0.027, p = 6.05) (**Figure 5A**). In addition, analysis using date retrieved from GEPIA database showed that SLC17A9 expression level was correlated with levels of immune marker genes of Tfh, Th1, Th17, M1 macrophage, M2 macrophage, Tumor associated macrophage (TAM), and natural killer cell (NK) cells (**Table 3**). Furthermore, TCGA LIHC analysis showed that SLC17A9 expression level was positively correlated with the infiltration level of CD4^+^ T cells and macrophages (**Figure 5B**). Patients were assigned to high and low expression groups to explore differences in immune infiltration levels under different SLC17A9 expression levels. The findings showed that infiltration of naive B cells (P = 0.015), naive CD4 T cells (P = 0.023), memory resting CD4 T cells (P = 0.033), T-cell regulatory (Tregs) cells (P = 0.036), and monocytes (P < 0.001) were significantly different between the high and low expression groups (**Figure 5C**).

**Table 3:** Correlation analysis between SLC17A9 and immune cell marker gene in GEPIA database.

Description	Gene Markers	GEPIA	
		Tumor	
		Correlation	P-Value
B cell	CD19	0.02	0.69
	MS4A1	−0.012	0.81
	CD79A	−0.028	0.57
CD8^+^ T cell	CD8A	−0.068	0.17
	CD8B	−0.048	0.32
	IL2RA	−0.03	0.53
Tfh	CXCR3	−0.009	0.85
	CXCR5	0.21	**2.20E-05**
	ICOS	−0.0026	0.96
Th1	IL12RB1	−0.065	0.18
	CCR1	−0.14	**0.0039**
	CCR5	−0.043	0.38
Th2	CCR4	0.00023	1
	CCR8	0.034	0.48
	IL21R	−0.045	0.36
Th17	IL23R	0.02	0.69
	CCR6	0.15	**0.0025**
	FOXP3	0.0098	0.84
Treg	NT5E	0.09	0.065
	IL7R	−0.019	0.7
	PDCD1	0.088	0.071
T-cell exhaustion	CTLA4	0.045	0.36
	LAG3	−0.024	0.62
	CD68	−0.049	0.32
M1 macrophage	ITGAM	−0.039	0.43
	NOS2	−0.0044	0.93
	IRF5	0.27	**2.30E-08**
M2 macrophage	CD163	−0.16	**0.00084**
	MRC1	−0.026	0.6
	CCL2	−0.1	**0.041**
TAM	CD86	−0.069	0.16
	CD14	−0.054	0.27
	CD33	−0.094	0.054
Monocyte	B3GAT1	−0.0096	0.84
	KIR3DL1	−0.085	0.081
	CD7	0.15	**0.0024**
Natural killer cell	FCGR3A	−0.094	0.054
	CD55	0.035	0.48
	CD1C	−0.0014	0.98
Neutrophil	THBD	−0.035	0.47
	CD19	0.02	0.69
	MS4A1	−0.012	0.81
Dendritic cell	CD79A	−0.028	0.57
	CD8A	−0.068	0.17
	CD8B	−0.048	0.32

Bold values indicate P < 0.05.

### Expression of SLC17A9 Is Correlated With m6A Methylation Regulator in LIHC

**Figure 6:**
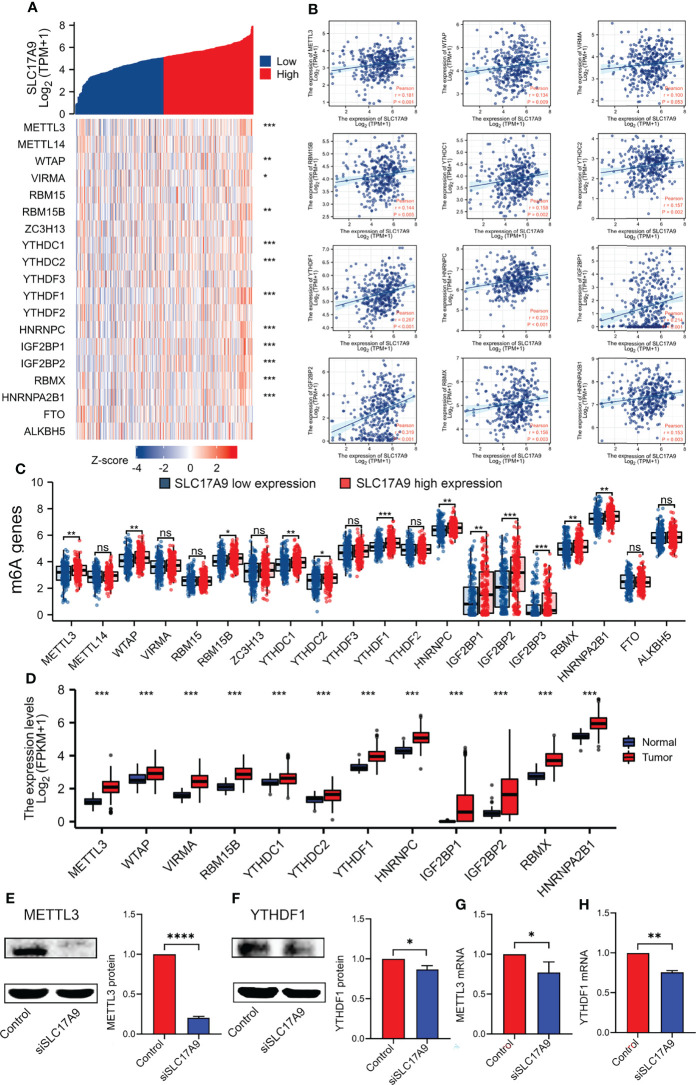
Correlations of SLC17A9 expression with m6A-related genes in LIHC. **(A)** Thermography shows the correlation between the expression of SLC17A9 and m6A-related genes. **(B)** The scatter plot shows the correlation between the expression of SLC17A9 and m6A-related genes. **(C)** Expression of m6A-related genes in the high and low SLC17A9 expression groups in LIHC. **(D)** m6A regulators expression in tumor and normal tissues. **(E, F)** METTL3 (left) and YTHDF1 (right) protein expression level in control and siSLC17A9 groups. **(G, H)** METTL3 (left) and YTHDF1 (right) mRNA expression level in control and siSLC17A9 groups.*p < 0.05; **p < 0.01; ***p < 0.001; ****p < 0.0001.

m6A modification is a potential target for designing drugs for tumor treatment. Therefore, the correlation between expression level of SLC17A9 and levels of m6A-related genes was explored using TCGA LIHC datasets (**Figures 6A, B**). The results showed that SLC17A9 expression was significantly correlated with level of METTL3 (r = 0.181, P < 0.001), WTAP (r = 0.134, P = 0.009), RBM15B (r = 0.144, P = 0.005), YTHDC1 (r = 0.158, P = 0.002), YTHDC2 (r = 0.157, P = 0.002), YTHDF1 (r = 0.267, P < 0.001), HNRNPC (r = 0.223, P < 0.001), IGF2BP1 (r = 0.214, P < 0.001), IGF2BP2 (r = 0.319, P < 0.001), RBMX (r = 0.156, P = 0.003), and HNRNPA2B1 (r = 0.153, P = 0.003) genes. The datasets were assigned into high and low expression groups to determine the expression differences between m6A modification–related genes at different SLC17A9 expression levels. The SLC17A9 high expression group showed significantly high expression levels of METTL3 (P = 0.006), WTAP (P = 0.003), RBM15B (P = 0.011), YTHDC1 (P = 0.006), YTHDC2 (P = 0.011), YTHDF1 (P < 0.001), HNRNPC (P = 0.001), IGF2BP1 (P = 0.002), IGF2BP2 (P < 0.001), IGF2BP3 (P < 0.001), RBMX (P = 0.009), and HNRNPA2B1 (P = 0.003) genes relative to the low expression group (**Figure 6C**). **Figure 6D** shows that m6A regulators expression change in tumor and normal tissues. On the basis of the above data, we screened two genes, METTL3 and YTHDF1, which not only increased expression in esophageal cancer, but also had the closest relationship with SLC17A9. Next, we used Western blots and RT-qPCR approaches to validate the functions of METTL3 and YTHDF1 genes at RNA and protein levels. The findings from Western blots showed significantly lower protein expression levels of METTL3 and YTHDF1 in the siSLC17A9 group compared with the expression levels in the control group (P < 0.0001, P = 0.03942; **Figures 6E** **, F**). RT-qPCR results showed significantly lower mRNA expression levels of METTL3 and YTHDF1 in the siSLC17A9 group compared with the expression levels in the control group (P = 0.038579, P = 0.002157; **Figures 6G** **, H**) .

### SLC17A9 Is Strongly Associated With Expression Levels of Iron Deficiency-Related Genes in LIHC

**Figure 7:**
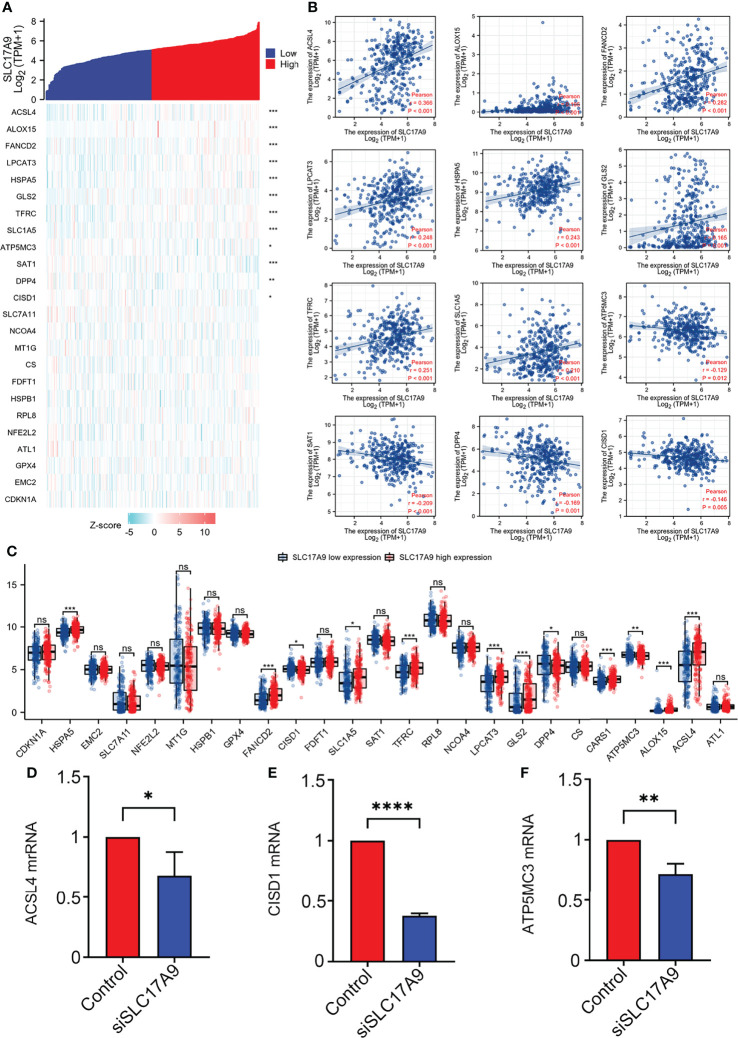
Correlations of SLC17A9 expression with Fe deficiency-related genes in LIHC. **(A)** Thermography shows the correlation between the expression of SLC17A9 and Fe deficiency-related genes. **(B)** The scatter plot shows the correlation between the expression of SLC17A9 and Fe deficiency-related genes. **(C)** Expression of Fe deficiency-related gene in the high and low SLC17A9 expression groups in LIHC. **(D)** ACSL4 mRNA expression level in control and siSLC17A9 groups. **(E)** CISD1 mRNA expression level in control and siSLC17A9 groups. **(F)** ATP5MC3 mRNA expression level in control and siSLC17A9 groups. *p < 0.05; **p < 0.01; ***p < 0.001; ****p <0.0001.

Further, the correlation between SLC17A9 and ferroptosis gene was explored using TCGA LIHC dataset (**Figures 7A, B**). The results showed that SLC17A9 expression level was significantly correlated with expression levels of HSPA5 (r = 0.240, p < 0.001), SLC7A11 (r = 0.180, p < 0.001), FANCD2 (r = 0.280, p < 0.001), CISD1 (r = 0.320, p < 0.001), SLC1A5 (r = 0.210, p < 0.001), AT1 (r = 0.210, p < 0.001), TFRC (r = 0.250, p < 0.001), LPCAT3 (r = 0.250, p < 0.001), GLS2 (r = 0.170, p = 0.001), DPP4 (r = 0.170, p = 0.001), ATP5MC3 (r = 0.130, p = 0.012), ALOX15 (r = 0.200, p < 0.001), and ACSL4 (r = 0.370, p < 0.001) genes. Moreover, the TCGA LIHC dataset was grouped into high and low expression groups to determine the expression difference in iron deficiency-related genes at different SLC17A9 expression levels. The findings showed that the expression levels of HSPA5 (p = 0.006), FANCD2 (p = 0.003), SLC1A5 (p = 0.011), SAT1 (p = 0.006), TFRC (p = 0.011), LPCAT3 (p < 0.001), GLS2 (p = 0.001), DPP4 (p = 0.002), CARS (p < 0.001), ATP5MC3 (p < 0.001), ALOX15 (p = 0.009), and ACSL4 (p = 0.003) were significantly higher in the SLC17A9 high expression group, relative to levels in the SLC17A9 low expression group (**Figure 7C**). ACSL4, CISD1, and ATP5MC3 genes were chosen for validation of these results using RT-qPCR analysis. The mRNA expression levels of ACSL4, CISD1, and ATP5MC3 in the siSLC17A9 group were significantly lower relative to the mRNA expression levels in the control group (P = 0.0481, P < 0.0001, P = 0.0048; **Figures 7D** **–F**).

## Discussion

SLC17A9 is a vesicle nucleotide transporter involved in ATP transport and is highly localized on lysosomes (25). Reduced SLC17A9 levels cause lysosomal ATP accumulation, lysosomal function impairment, and cell death. Mounting evidence has associated the lysosome with cancer. During tumorigenesis, the lysosome location and lysosomal membrane permeability change, releasing vast amounts of cathepsin into the cytoplasm, which, together with matrix metalloproteinases and blood plasminogen activation system, promotes tumorigenesis by degrading the extracellular matrix. Thus, SLC17A9 expression directly or indirectly affects tumorigenesis. Past studies have implicated SLC17A9 upregulation in gastric (26), liver (27), and colon cancer (28). However, its expression and function in liver cancer have not been determined. Using IHC, we show that SLC17A9 upregulated in LIHC and further knocked out the SLC17A9 gene for *in vitro* experiments. We find that SLC17A9 silencing HepG2 markedly suppressed ATP production as well as cell proliferation, migration, and colony formation. In LIHC, SLC17A9 expression significantly correlated with sex, DSS, and PFI, indicating that it may have prognostic value in LIHC. ROC curve analysis highlighted the diagnostic value of SLC17A9 expression in LIHC. Second, we find that patients with LIHC expressing high SLC17A9 levels had worse prognosis than those with low SLC17A9 levels, indicating that SLC17A9 has potential diagnostic and prognostic value in LIHC.

KEGG pathway enrichment analysis showed that SLC17A9 expression was mainly associated with PI3K/Akt signaling pathway, neuroactive ligand-receptor interaction, HPV infection, MAPK signaling, passive transmembrane transporter activity, and channel activity. It is reported that LLGL2 drives carcinogenic PI3K/AKT signaling in LIHC to promote LIHC (29). In addition, TMOD3 thought to promote liver cancer progression *via* MAPK/ERK signaling (30). These metabolic pathways can directly or indirectly affect tumorigenesis and highlight potential multi-target and multi-pathway synergistic treatment for LIHC. We speculate that SLC17A9 may interact with these pathways to promote liver cancer progression.

GO term enrichment analysis showed that SLC17A9 expression is associated with many aspects of BP, CC, and MF and are mainly associated with neuronal cell body, presynapse, and axonogenesis, indicating that SLC17A9 may be involved in protein binding, plasma membrane, membrane composition, nucleus, and other biological functions.

GGI and PPI analysis of co-expressed genes revealed the strongest correlation to be between MYO5A, SLC18A3, and SLC17A9. MYO5A is a key component of the myosin V family, which participates in transport vesicle formation, protein transcription, and tumor progression (31). Studies have implicated MYO5A in laryngeal squamous cell carcinoma and esophageal squamous cell carcinoma (32, 33). SLC18A3 belongs to the family of vesicular acetylcholine transporters. Various acetylcholine signaling pathways can regulate a range of cellular functions, including proliferation, differentiation, and cytoskeleton integrity (34). L cells from small cell lung cancer are reported to secrete acetylcholine and promote tumor proliferation (35). These results suggest that the expression of MYO5A and SLC18A3 affects tumorigenesis, which provide a theoretical basis in treatment of liver cancer.

Analysis of the relationship between SLC17A9 and immune cell infiltration in LIHC revealed that SLC17A9 expression correlates with B cells, CD4^+^ T cells, macrophages, neutrophils, and immune marker genes for Tfh, Th1, Th17, M1 macrophage, M2 macrophage, TAM, and NK cells. It was also positively associated with the degree of infiltration by infiltration CD4^+^ T cells and macrophages. CiberSort analysis showed that naive B cells, naive CD4^+^ T cells, memory resting CD4^+^ T cells, regulatory T cells (Tregs), and monocytes differed significantly with SLC17A9 expression. Together, these results confirmed that SLC17A9 was associated with tumor infiltration by immune cells in LIHC, especially B cells, CD4^+^ T cells, and macrophages. Macrophages have been associated with immunosuppression and angiogenesis, which provides sufficient nutrition for tumor proliferation (36). SLC17A9 expression and vesicular ATP exocytosis by macrophages can activate the P2 receptors and participate in macrophage activation expression (37). We speculate that high SLC17A9 expression in LIHC may activate macrophages, which influence LIHC proliferation.

m6A methylation is the most common mRNA modification in eukaryotes, and it influences proliferation and migration of tumors. Here, analysis on various databases found that SLC17A9 expression positively correlated with METTL3, YTHDF1, and WTAP expression. Western blot analysis and RT-qPCR analysis revealed that siSLC17A9 silencing reduced the expression of METTL3 and YTHDF1 significantly relative the control group. RNA-seq analysis revealed that HNNPC was specifically upregulated in LIHC. It is reported that METTL3 overexpression promotes LIHC growth *in vivo* and *in vitro* (38) and that this correlates with poor prognosis in LIHC. On the basis of these findings, we point out that the SLC17A9 modification by m6A enhances its mRNA stability, thereby promoting LIHC growth.

Iron cell death is reported to play an important role in tumorigenesis. Here, database analysis showed that SLC17A9 expression correlated with the expression of various genes, including lACSL4, CISD1, and ATP5MC3. These observations were verified by RT-qPCR analysis, which showed that ACSL4, CISD1, and ATP5MC3 expression was significantly lower in siSLC17A9 cells relative to the controls. ACSL4 is an enzyme that regulates lipid composition. It can also promote ferroptosis. It is reported that ACSL4 promotes LIHC *via* the ERK/FBW7/c-Myc axis (39). Numerous studies have reported CISD1 expression in LIHC. ATP5MC3 has been associated with colon cancer (40). We speculate whether SLC17A9 interaction with other genes like ACSL4, CISD1, and ATP5MC3 affects iron-dependent cell death in LIHC.

The core of our article is to discuss the expression of SLC17A9 in hepatocellular carcinoma and further transfect siRNA to verify it by *in vivo* experiments. Some information related to immune infiltration, m6A, and iron death were added, and several m6A-related factors with strong correlation were screened to verify the protein expression level and RNA expression level. Our results do show that the expression levels of METTL3 and YTHDF1 in the siSLC17A9 group are lower than those in the control group, but our results are only superficial and only validate individual factors, and no batch in-depth research has been done, which is one of our defects and deficiencies.

To our knowledge, this is the first study to comprehensively analyze SLC17A9 expression in LIHC with regards to cellular function. SLC17A9 upregulation in LIHC correlates with poor prognosis and has a significant correlation with biology, highlighting its potential therapeutic and diagnostic potential.

## Data Availability Statement

The original contributions presented in the study are included in the article/supplementary materials. Further inquiries can be directed to the corresponding author.

## Ethics Statement

The studies involving human participants were reviewed and approved by Ethics Committee of Taihe Hospital Affiliated of Hubei University of Medicine. Written informed consent for participation was not required for this study in accordance with the national legislation and the institutional requirements.

## Author Contributions

X-YK and YG conceived the project and wrote the manuscript. X-YK, JZ, J-WY, and X-SL participated in data analysis. X-YK, YG, L-MZ, and X-YL participated in the discussion and language editing. Z-JP reviewed the manuscript. All authors contributed to the article and approved the submitted version.

## Funding

This work was supported by the Hubei Province’s Outstanding Medical Academic Leader program, the Foundation for Innovative Research Team of Hubei Provincial Department of Education (No. T2020025), the general project of Hubei Provincial Department of Education (No. B2021160), the HuBei Provincial Department of Science and Technology Innovation Group Programme (grant no. 2019CFA034), Innovative Research Program for Graduates of Hubei University of Medicine (grant nos. YC2020011 and YC2021018), and the Key Discipline Project of Hubei University of Medicine.

## Conflict of Interest

The authors declare that the research was conducted without any commercial or financial relationships that could be construed as a potential conflict of interest.

## Publisher’s Note

All claims expressed in this article are solely those of the authors and do not necessarily represent those of their affiliated organizations, or those of the publisher, the editors and the reviewers. Any product that may be evaluated in this article, or claim that may be made by its manufacturer, is not guaranteed or endorsed by the publisher.
